# A Generalized Structural Equation Model Approach to Long Working Hours and Near-Misses among Healthcare Professionals in Japan

**DOI:** 10.3390/ijerph18137154

**Published:** 2021-07-04

**Authors:** Tatsuhiko Anzai, Takashi Yamauchi, Masaki Ozawa, Kunihiko Takahashi

**Affiliations:** 1 Department of Biostatistics, M&D Data Science Center, Tokyo Medical and Dental University, Tokyo 113-8510, Japan; tanzai.dsc@tmd.ac.jp; 2 Department of Public Health and Environmental Medicine, The Jikei University School of Medicine, Tokyo 105-8461, Japan; yamauchi-t@jikei.ac.jp; 3 School of Medicine, Tokyo Medical and Dental University, Tokyo 113-8510, Japan; 170211ms@tmd.ac.jp

**Keywords:** near-miss, generalized structural equation model, healthcare professional, indirect effect

## Abstract

(1) Background: Near-miss incidents are the foundation of major injuries. They are warning signs that loss is imminent. Long working hours are a risk factor for near-misses along with sleep problems, job-related stress, and depressive symptoms. This study aimed to evaluate the indirect effects of long working hours via mediating variables on near-miss occurrences among Japanese healthcare professionals. (2) Methods: 1490 Japanese healthcare professionals’ reports from a web-based survey of workers in October 2018 were analyzed to evaluate total, direct, and indirect effects of long working hours on near-misses. We applied a generalized structural equation model with three mediating variables: sleep problems, job-related stress, and depressive symptoms. (3) Results: The total effect and direct effect of the categories of working hours longer than 41 h per week (h/w) for occurrence of near-misses were not significantly higher than that of 35–40 h/w. However, for indirect effects on occurrence of near-misses that first passed through job-related stress, there were higher reports for each category compared to 35–40 h/w, with odds ratios (OR) and 95% confidence intervals (95% CI) of OR = 1.12, 95% CI (1.07, 1.21) for 41–50 h/w; 1.25, (1.14, 1.41) for 51–60 h/w; and 1.31, (1.18, 1.51) for ≥ 61 h/w. (4) Conclusion: The results suggest that reducing working hours might improve job-related stress, which could reduce near-misses and prevent injuries.

## 1. Introduction

Many healthcare professionals interact with and treat patients in a variety of situations daily [1,2]. Occupational injuries among healthcare professionals have a notable impact not only on the professionals themselves but also on the lives of patients. Recent reports, in general, have suggested that “near-miss” incidents (i.e., “an unplanned event that did not result in injury, illness, or damage but had the potential to do so”) [3] are the foundation of major injuries and are warning signs that loss is imminent [4]. Alamgir et al. mentioned a similar causal relationship between near-misses and injuries, including serious injuries and deaths [5]. Yamauchi et al. reported that near-misses and minor injuries precede severe injuries in industrial incidents, and thus can predict severe injuries [6]. Among healthcare professionals, near-misses can be viewed as “an error in care that has substantial potential to cause harm but does not, either because it is intercepted or because it unexpectedly causes no apparent harm despite reaching the patient” [7]. Such medical errors, including several types of medication errors [5], may lead healthcare providers to reassess their clinical skills, knowledge base, or career choice [8].

Previous research has suggested that long working hours are one of the factors increasing the risk of near-misses [9,10,11]. Studies on the effect of long working hours on near-misses have shown that working more than 40 h per week (h/w) increased the risk of near-misses relative to workers who had shorter working hours in US nurses [9]. Among workers in the medical, health, and welfare industry in Japan, those who worked 40–51 h/w experienced more near-misses than those who worked 35–40 h/w. [10] Long working hours are more prevalent in Japan than in developed Western countries, including for healthcare workers [12,13,14]. Among Japanese healthcare professionals, there are concerns about an increase in long working hours and a decrease in rest periods related to the coronavirus disease pandemic [15] and increased population aging [16], among other concerns.

Meanwhile, several epidemiological studies have shown the negative effects of long working hours on the risks of chronic fatigue, stress, depressive state, anxiety, and sleep quality [17,18]. These factors also increase the risk of near-misses and may mediate the effects of long working hours on near-misses [19]. A previous study suggested that the effects of working hours on injuries and near misses among Korean construction workers are affected indirectly by the mediating effects of physical or psychological symptoms/strain [20]. However, the direct and indirect effects of long working hours on near-misses and the potential mediating influence of job-related stress, sleep problems, and depressive symptoms have not been assessed for Japanese healthcare professionals.

Hence, this study evaluated the relationship between long working hours and near-misses, including the indirect influence through the medium of the status of healthcare professionals in Japan. In particular, we assessed the direct effect of long working hours related to occurrence of near-misses and the indirect effects of long working hours, taking into consideration the mediators of job-related stress, sleep problem, and depressive symptoms. In our analyses, we applied a structural equation model (SEM), which is often used to address similar issues in such fields as psychology and social epidemiology [21]. In particular, we employed a generalized structural equation model (GSEM) to model the binary responses [22]. This analysis shows the impact of working hours for each mediated variable and it could help develop measures to promote the prevention of occupational injury.

## 2. Materials and Methods

### 2.1. Participants and Measures

This study conducted a web-based cross-sectional survey of workers in October 2018 in collaboration with a research company that has one of the largest research panels in Japan, with over 1.8 million voluntarily registered panelists [10]. To minimize selection bias, the survey randomly selected registered Japanese workers aged 20–64 years based on the composition ratio of workers by industry, sex, and age in the 2017 Labour Force Survey. The research company randomly sent an e-mail invitation for participation in the study to registered workers. Workers who provided web-based informed consent were selected to participate in the survey; they answered a self-rated questionnaire on the internet, which consisted of questions regarding demographic, job-related, and life-related variables as well as health/safety-related outcomes. The research company recruited participants until the total number of participants who completed the web-based questionnaire reached 30,000 workers.

We selected full-time employees (over 35 working h/w as the average working hours during the last six months) who reported being professional, technical experts, or researchers in the medical/health/welfare industry based on the Japan Standard Industrial Classification established by the Japanese Ministry of Internal Affairs and Communications [23].

The outcome measures of the present study were near-misses, which were defined as events that did not result in but had the potential to cause injury in industrial settings during the past six months. In this survey, this definition of a near-miss was presented as “an unplanned event that did not result in injury, illness, or damage but had the potential to do so,” after which the participants were asked whether they had experienced any near- misses in industrial settings with the following question: “Have you experienced any near-misses in a workplace setting during the past six months?”

Self-reported working h/w during the past six months were grouped into the following categories: 35–40 h/w, 41–50 h/w, 51–60 h/w, and ≥61 h/w. To represent the mediated variables of long working hours, we evaluated overall sleep quality, depressive symptoms, and job-related stress. We used the Pittsburgh Sleep Quality Index (PSQI) to assess overall sleep quality [24]. The PSQI is an 18-item self-reported questionnaire designed to assess overall sleep quality during the past month and demonstrates favorable psychometric properties. In the present study, we used the Japanese version of the PSQI. The total score ranges from 0 to 21 [25]. To measure depressive symptoms, we used the Japanese version of the Center for Epidemiological Studies Depression Scale (CES-D) [26]. The CES-D is a 20-item self-rated questionnaire that measures depressive symptoms during the past week and shows acceptable reliability and validity. Total scores range from 0 to 60, with higher scores indicating greater severity of depressive symptoms. To assess job-related stress, we used the “job-related stressors” from the Brief Job Stress Questionnaire (BJSQ), which has been widely used to measure job-related stress in Japan [27]. Job stressors include qualitative job burden, quantitative job burden, job control, and interpersonal conflict (17 items). Total scores range from 17 to 68. The study protocol was approved by the Institutional Review Board of the Jikei University School of Medicine, Tokyo, Japan, in 2018 (No. 30-153(9174)).

### 2.2. Structure Model

**Figure 1:**
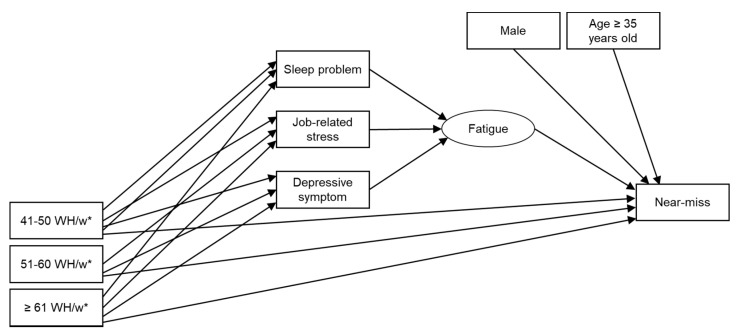
Structural equation model for the relationship between near-misses and working hours. WH, working hours; w, week. * Reference category is 35–40 WH/w. “41–50 WH/w”, “51–60 WH/w”, “≥61 WH/w”, “Male”, and “Age ≥ 35 years old” are dummy variables, coded 1 if applicable and 0 otherwise; the circle represents a latent variable.

The items of job-related stress, sleep problems, and depressive symptoms were included as mediating variables in the structural model of near-misses and working hours. A simple model was used to separate the direct effect of working hours, which was the main objective, from the indirect effect of the mediating variables. The effects of long working hours discussed in previous studies suggest that these mediating variables can cause mental or physical fatigue [12,16,27]. There may be concern that these mediating variables have more complex interrelationships, which we tried to take into account by including the latent variable as composite variables of mediating variables in the model for simple evaluation. Therefore, the factor of fatigue was included in the model as a latent variable in this study. Figure 1 shows the model with the minimum confounding factor of risk of near-misses.

In addition, for our sensitivity analysis of model structure, we employed a model that adds a modifier, such as the four-scaled recovery score of fatigue and the presence or absence of night/shift work (see Figure S1a,b). Furthermore, as another sensitivity analysis to evaluate the impact of reflecting relationships among mediating variables on the results, we assessed the effects using a model in which the paths from job-related stress to sleep problems and from job-related stress to depression were added.

### 2.3. Statistical Analysis

Demographics and baseline characteristics were summarized. We employed GSEM to estimate the direct and indirect effect of each category of long working hours relative to 35–40 h/w on near-misses. GSEM procedures have been developed to allow some types of variables, including binary data, and recently have been used for mediation analysis [28]. In this study, bootstrap resampling was used to obtain 95% confidence intervals (95% CI) for these effects, including the mediation effect, based on the percentile method. In the model analysis based on GSEM, a statistical significance was defined at the 95% CI for difference measures that did not include the value of 0.

Furthermore, we conducted a subgroup analysis to assess the difference in the response to working hours on near-misses between novice and experienced workers among younger age groups. We categorized healthcare professionals aged under 35 years into two groups: those with ≤3 years or >3 years of current work experience, and applied the model for each.

Statistical analysis was performed using STATA version 16 (STATA Corp, College Station, TX, USA) and R software, version 4.0.4 (R Core Team).

## 3. Results

From the voluntarily registered panelists, 30,000 participants for the survey were randomly selected. Among all job types of participants, 2028 healthcare professionals were identified. We analyzed the reports of 1490 healthcare professionals with over 35 working h/w as the average working hours during the last six months of service at the time of the survey and with responses for the presence or absence of near-misses.

The characteristics of healthcare professionals are shown in Table 1. Among the healthcare professionals, 529 participants (35.5%) were between 20 and 34 years old, and 1,143 (76.7%) were female. Overall, 732 healthcare professionals (49.1%) reported the presence of near-misses.

Table 2 shows the estimated coefficients of the GSEM in Figure 1. Binary responses and odds ratios (ORs) of reports of near-misses compared to the reference group are also shown by exponentially transforming the coefficients. The coefficients for working hour categories longer than 41 h/w on near-misses (i.e., direct effect) were not statistically significant when the group of 35–40 h/w is the reference. The coefficients of job-related stress, sleep problems, and depressive symptoms tended to increase with longer working hours. The effect of mediating variables on fatigue were positive, except for depressive symptoms.

Table 3 shows the direct, indirect, and total effects of working hours on near-misses. For the total effect, the ORs for near-misses for each working hour category relative to 35–40 h/w were 1.26, 95% CI (0.97, 1.61) for 41–50 h/w; 1.41, 95% CI (0.99, 2.06) for 51–60 h/w; and 1.41, 95% CI (0.96, 2.06) for ≥61 h/w and they did not reach statistical significance. Meanwhile, for the direct effect, the ORs for near-misses for the working hour categories relative to 35–40 h/w were 1.11, 95% CI (0.85, 1.41) for 41–50 h/w; 1.09, 95% CI (0.77, 1.60) for 51–60 h/w; and 1.02. 95% CI (0.69, 1.48) for ≥61 h/w. For the indirect effect, the ORs for near-misses that first passed through job-related stress were observed as statistically significant relative to the 35–40 h/w category (41–50 h/w: OR = 1.12, 95% CI (1.07, 1.21); 51–60 h/w: OR = 1.25, 95% CI (1.14, 1.41); and ≥61 h/w: OR = 1.31, 95% CI (1.18, 1.51)). Additionally, for indirect effects, the ORs for near-misses that first passed through sleep problems were 1.01, 95% CI (0.99, 1.05) for 41–50 h/w; 1.04, 95% CI (1.00, 1.10) for 51–60 h/w; and 1.07, 95% CI (1.02, 1.13) for ≥61 h/w relative to the 35–40 h/w category. Meanwhile, the ORs of indirect effects of working hours on near-misses that first passed through depressive symptoms were about 1.00 relative to the 35–40 h/w for all working hour categories.

In addition, the results of the sensitivity analysis for the model structure, which added the recovery score of fatigue and the presence or absence of night/shift work, showed that the direct and indirect effects on near-misses of each working hour category did not change substantially from the main analysis (see Table S1). However, total effects for each working hour category were slightly statistically significant. The results of another analysis based on the model including the relationships between each intermediate variable showed that only the indirect effects of the path through job-related stress and sleep problems were statistically significant, but these ORs were very close to 1.00 (1.02 to 1.05) (Table S2). As for the total effect, only the category of 51–60 h/w showed slightly statistically significant results. There is no change in the results of the direct effects of working hour on near-misses through our sensitivity analyses.

Lastly, the results of the subgroup analysis by category of work experience for healthcare professionals are shown in Table 4. The ORs of direct effects for long working hours on near-misses were not statistically significant for both subgroups. For the indirect effects, the ORs that first passed through sleep problems for each working hour category on near-misses were 1.04, 95% CI (0.89, 1.24) for 41–50 h/w; 1.35, 95% CI (1.01, 2.07) for 51–60 h/w; and 1.40, 95% CI (1.02, 2.42) for ≥61 h/w in the subgroup of ≤3 years of work experience. Meanwhile, working hours might not be related to the larger number of near-misses in the subgroup with >3 years of work experience. In the column showing the total effect, for the subgroup with ≤3 years of work experience, the category of ≥61 h/w of working hours has the highest OR for near-misses.

## 4. Discussion

In the present study, we examined the association between long working hours and near-misses among Japanese healthcare professionals using a nationally representative sample of healthcare workers with respect to sex and age. Near-misses were reported in 49% of the participants in this study. Near-misses related to the administration of medicines were reported in about 10 to 20% of the study population [29], and the proportion of such near-misses were only 30% of all near-misses [30]. The analysis of near-misses forms part of the health and safety management systems of various organizations to manage occupational health and safety risks [31], including the healthcare industry. Specifically, near-miss incidents, regardless of their minor or major consequences, have been found to be a critical metric of a health and safety management system as well as “free lessons for safety management” [32].

Previous research on the broader medical/health/welfare industry not limited to healthcare professionals showed that the OR for near-misses for the working hour category of 41–50 h/w was 1.20 with statistical significance relative to 35–40 h/w; however, for the ≥61 h/w category, the OR for near-misses was closer to 1.00 [10]. In our study, the total effect for all categories of working hours for healthcare professionals tended to be related to the occurrence of near-misses relative to the category 35–40 h/w, which did not reach statistical significance in our model but was slightly significant in the models taking into account such factors as recovery from fatigue or more complex model structures. Other than this difference of a slight statistical significance in the total effects among models, there were no changes in each direct or indirect effect. Nevertheless, more detailed adjustment analysis is needed to confirm whether there is a significant difference in total effects. The point estimates of ORs for all categories of working hours above 41 h/w are almost the same across these models, and the values are larger than 1.20, which may indicate that working hours longer than 41 h have a total effect of more near-misses. In addition, although statistical significance may vary owing to the division into subpopulations, the risk of near-misses was higher among healthcare workers with less experience in their current jobs, which is consistent with previous studies [29,33]. However, a relationship between long working hours and near-misses was also found among those with more experience. The results suggest that decreasing working hours may reduce near-misses regardless of work experience.

Based on our model, decomposing the impact of working hours on near-misses into direct and indirect effects showed that the direct effects of long working hours were not significant. Most of the impact of long working hours on near-misses is the path that first passed through job-related stress. Stressors such as workload, time pressure, and understaffing, which may be related to long working hours, have been shown to influence near-misses [29], and these may be reflected in job-related stress [20]. The results show that working hours were positively related to job-related stress, with the latent variable of fatigue having the strongest association in our model. Long working hours may lead to decreased concentration and attention through increased fatigue. Previous systematic reviews have suggested that fatigue detrimentally affects job performance, decision-making, and, subsequently, safety [34,35]. Many healthcare professionals must interact with patients in various situations daily, even when under a heavy workload or experiencing high stress. Highly stressed healthcare professionals might be at an increased risk of commission errors for their patients [36]. Research evaluating the relationship between the types of errors in high-stress situations among healthcare professionals might be beneficial.

In addition, the variables having an indirect effect through job-related stress may provide information on what to monitor regarding the role of long working hours on near-misses because the greatest influence of long working hours on near-misses is the indirect path that first passes through job-related stress. The Japanese Industrial Safety and Health Law was expanded in 2015 to include a stress check program that mandates annual screenings of high psychosocial stress workplaces for enterprises with 50 or more employees using the BJSQ [27,37]. Thus, many medical workplaces in Japan can monitor the risk of near-misses without introducing a new measure. Exploring and validating the appropriate thresholds for these scores might be necessary to predict near-misses.

Inadequate recovery from sleep problems is an important component of the pathway from long working hours to increased fatigue and the risk of health problems [38,39]. Our results are consistent with those of previous studies [40], reporting that sleep worsens with working hours, although the effects are smaller than those of job-related stress. In addition, the effects of sleep on fatigue appear smaller than those of job-related stress. The difference in the percentage of sleep problems between the group with long working hours and that with regular working hours was about 10% [39]. Thus, the effects of working hours leading to near-misses might not be large. For example, a relationship between near-misses and drowsiness among drivers has been observed [41], but the effect of working hours on near-misses via sleep problems may differ by occupation.

Among the workers in a previous study that included healthcare professionals, depression significantly increased the ORs for near-misses [10]. However, in our study of healthcare professionals, the indirect influence of long working hours on near-misses through depressive symptoms was initially limited. Long working hours ≥ 61 h/w could significantly affect depressive symptoms. Although reducing the working hours of healthcare professionals might decrease depressive symptoms, it might not decrease near-misses through changes in depressive symptoms. One reason may be that depressive symptoms are related to physical inactivity [42]. Since near-misses among healthcare professionals might occur during patient interactions, physical inactivity might not be related to an increase in near-misses.

This approach of disentangling the indirect and direct effects could provide information on preventive measures other than reducing long working hours, not only for healthcare professionals. Our result suggests that interventions targeting work-related stressors and positive coping strategies (e.g., looking for support from others, utilizing others’ ways of dealing with similar problems), which reduce the negative effects of work stress on job performance [43], might prevent near-misses for healthcare professionals in Japan. Further research could help find effective interventions to prevent near-misses and future injuries at work by occupation.

Our study has several limitations. First, we relied on self-reported job-related factors, including working hours, for exposure and near-misses as the outcome measure, which might have been influenced by recall bias. Since this is a web-based survey, there is a possibility that the selection of the respondents may be biased. Second, various confounding individual and environmental factors were not available, such as demographics and working conditions among healthcare professionals. Therefore, this study did not examine the effect of reducing working hours on the occurrence of near-misses. Third, the participants’ reports might not represent the actual occurrence of near-misses, because detecting and reporting them could depend on several factors, such as the experience of current work [29]. However, reporting of near-misses and accidents is an important learning system in medicine [44], so the quality of reporting to prevent injuries must be maintained. Fourth, differences in the association between working hours and near-misses/injuries by occupation (i.e., physicians, nurses, and elderly care workers) in the healthcare industry may exist. Fifth, in studies using SEM, several indicators of model fit are assessed; however, none of these traditional models fit indicators can be applied to models employing logistic regression analysis with maximum likelihood estimation [45]. Although a sensitivity analysis was conducted to account for the recovery score from fatigue and night shifts, the estimation of the effect of working hours was similar to that in the main analysis. Another sensitivity analysis based on the model including associations between mediator variables did not change the conclusions.

## 5. Conclusions

This study evaluated the direct and indirect effect of long working hours on near-misses among Japanese healthcare professionals. Long working hours are associated with the occurrence of near-misses through job-related stress and sleep problems. Therefore, reducing working hours might improve the job-related stress levels of healthcare professionals, which might reduce near-misses and prevent injuries. To reduce the number of near-misses, it may be important to examine the effect of reducing working hours or to examine the effect of intervention on the intermediate variable, job-related stress.

## Figures and Tables

**Table 1:** Demographics and characteristics of healthcare professionals at the time of the survey.

	Healthcare Professionals (*N* = 1490)
Female, *n* (%)	1143 (76.7)
Age, *n* (%)	
20–34 years	529 (35.5)
35–64 years	961 (64.5)
Work hours per week, *n* (%)	
35–40 h/w	507 (34.0)
41–50 h/w	665 (44.6)
51–60 h/w	170 (11.4)
≥61 h/w	148 (9.9)
Night/shift work, *n* (%)	553 (37.1)
Work experience, *n* (%)	
≤3 years	283 (19.0)
4–10 years	481 (32.3)
≥11 years	726 (48.7)
Job-related stress (total score), mean (SD)	44.0 (6.3)
Depressive symptom (total score), mean (SD)	16.5 (9.7)
Sleep problem (total score), mean (SD)	5.8 (3.0)
Near-miss, *n* (%)	732 (49.1)

h/w, hours per week; SD, standard deviation.

**Table 2:** Estimated standardized coefficients and odds ratios of generalized structural equation model for near-misses during the past six months among healthcare professionals.

	Standardized Coefficient (95% CI)	Odds Ratio ^1^ (95% CI)
Working hours → Near-misses		
35–40 h/w	reference	1.00
41–50 h/w	0.100 (−0.153, 0.346)	1.11 (0.85, 1.41)
51–60 h/w	0.087 (−0.261, 0.467)	1.09 (0.77, 1.60)
≥61 h/w	0.016 (−0.375, 0.394)	1.02 (0.69,1.48)
		
Working hours → Job-related stress		
35–40 h/w	reference	-
41–50 h/w	0.290 (0.176, 0.404)	-
51–60 h/w	0.556 (0.382, 0.728)	-
≥61 h/w	0.690 (0.524, 0.859)	-
		
Working hours → Sleep problem		
35–40 h/w	reference	-
41–50 h/w	0.056 (−0.063, 0.171)	-
51–60 h/w	0.168 (−0.019, 0.349)	-
≥61 h/w	0.263 (0.082, 0.442)	-
		
Working hours → Depressive symptom		
35–40 h/w	reference	-
41–50 h/w	0.007 (−0.108, 0.126)	-
51–60 h/w	0.142 (−0.021, 0.297)	-
≥61 h/w	0.362 (0.139, 0.580)	-
		
Job–related stress → Fatigue (latent variable)	0.397 (0.278, 0.532)	-
Sleep problem → Fatigue (latent variable)	0.239 (0.114, 0.365)	-
Depressive symptom → Fatigue (latent variable)	−0.021 (−0.166, 0.121)	-
		
Fatigue (latent variable) → Near–misses	1.000 ^2^	-
		
Sex → Near–misses		
Female	reference	1.00
Male	0.070 (−0.179, 0.311)	1.07 (0.84, 1.37)
		
Age → Near–misses		
< 35 years old	reference	1.00
≥35 years old	−0.157 (−0.378, 0.072)	0.85 (0.68, 1.07)

95% CI, 95% confidence interval; h/w, hours per week. ^1^ Odds ratios were derived for binary responses. ^2^ Fixed as 1.00 for scale settings.

**Table 3:** Direct, indirect, and total effects of working hours on near-misses during the past six months for healthcare professionals.

	n/Number of Near-Misses	Direct Effect	Indirect Effect			Total Effect (Direct + Indirect)
		Working Hour → Near-Miss	Working Hour → Job-Related Stress → Fatigue → Near-Miss	Working Hour → Sleep Problem → Fatigue → Near-Miss	Working Hour → Depressive Symptom → Fatigue → Near-Miss	
		OR (95%CI)	OR (95%CI)	OR (95%CI)	OR (95%CI)	OR (95%CI)
Working hours/week						
35–40 h/w	507/231	1.00 (reference)	1.00 (reference)	1.00 (reference)	1.00 (reference)	1.00 (reference)
41–50 h/w	665/338	1.11 (0.85, 1.41)	1.12 (1.07, 1.21)	1.01 (0.99, 1.05)	1.00 (0.99, 1.01)	1.26 (0.97, 1.61)
51–60 h/w	170/92	1.09 (0.77, 1.60)	1.25 (1.14, 1.41)	1.04 (1.00, 1.10)	1.00 (0.97, 1.02)	1.41 (0.99, 2.06)
≥61 h/w	148/80	1.02 (0.69, 1.48)	1.31 (1.18, 1.51)	1.07 (1.02, 1.13)	0.99 (0.93, 1.05)	1.41 (0.96, 2.06)

OR, odds ratio; 95% CI, 95% confidence interval; h/w, hours per week.

**Table 4:** Direct, indirect, and *total* effects of working hours on near-misses during the past six months by work experience subgroup among healthcare professionals aged under 35 years.

	*n*/Number of Near-Misses	Direct Effect	Indirect effect			Total Effect (Direct + Indirect)
		Working Hour → Near-Miss	Working Hour → Job-Related Stress → Fatigue → Near-Miss	Working Hour → Sleep Problem → Fatigue → Near-Miss	Working Hour → Depressive Symptom → Fatigue → Near-Miss	
		OR (95%CI)	OR (95%CI)	OR (95%CI)	OR (95%CI)	OR (95%CI)
Work experience ≤ 3 years (*N* = 203)						
Working hours/week						
35–40 h/w	60/28	1.00 (reference)	1.00 (reference)	1.00 (reference)	1.00 (reference)	1.00 (reference)
41–50 h/w	92/53	1.37 (0.66, 2.85)	1.16 (1.00, 1.47)	1.04 (0.89, 1.24)	1.01 (0.94, 1.14)	1.67 (0.77, 3.66)
51–60 h/w	34/21	1.22 (0.45, 3.57)	1.26 (1.00, 1.90)	1.35 (1.01, 2.07)	1.03 (0.90, 1.21)	2.14 (0.76, 6.48)
≥61 h/w	17/12	1.39 (0.39, 8.26)	1.52 (1.00, 2.90)	1.40 (1.02, 2.42)	1.06 (0.81, 1.45)	3.14 (1.04, 26.66)
Work experience > 3 years (*N* = 326)						
Working hours/week						
35–40 h/w	109/45	1.00 (reference)	1.00 (reference)	1.00 (reference)	1.00 (reference)	1.00 (reference)
41–50 h/w	129/69	1.49 (0.88, 2.68)	1.10 (1.01, 1.27)	1.01 (0.96, 1.09)	1.02 (0.96, 1.12)	1.68 (0.97, 3.18)
51–60 h/w	41/23	1.48 (0.68, 3.36)	1.17 (1.02, 1.47)	1.02 (0.94, 1.15)	1.05 (0.96, 1.20)	1.85 (0.85, 4.31)
≥61 h/w	47/27	1.50 (0.72, 3.18)	1.22 (1.04, 1.54)	1.02 (0.93, 1.16)	1.08 (0.98, 1.29)	2.00 (0.95, 4.37)

OR, odds ratio; 95% CI, 95% confidence interval; h/w, hours per week.

## Data Availability

Data cannot be shared publicly, as they contain information that could compromise the privacy of research participants based on the “Ethical Guidelines for Medical and Health Research involving Human Subjects” by the Japanese government.

## Supplementary Materials

The following are available online at https://www.mdpi.com/article/10.3390/ijerph18137154/s1: Figure S1—Structural equation model for relationship between near-misses and working hours in sensitivity analysis; Table S1—Direct, indirect, and total effects of working hours on near-misses for sensitivity analysis; and Table S2—Direct, indirect, and total effects of working hours on near-misses during the past six months in healthcare professionals for model including relationship among the mediated variables.
