# Supplementary material for: A Generalized Structural Equation Model Approach to Long Working Hours and Near-Misses among Healthcare Professionals in Japan
Source: Int J Environ Res Public Health. 2021 Jul 4;18(13):7154. doi: 10.3390/ijerph18137154 (PMC8296918; doi:10.3390/ijerph18137154)
Supplement: Supplementary file 1 [file ijerph-18-07154-s001.zip › supp_figure_p.pdf]

a: Model 2

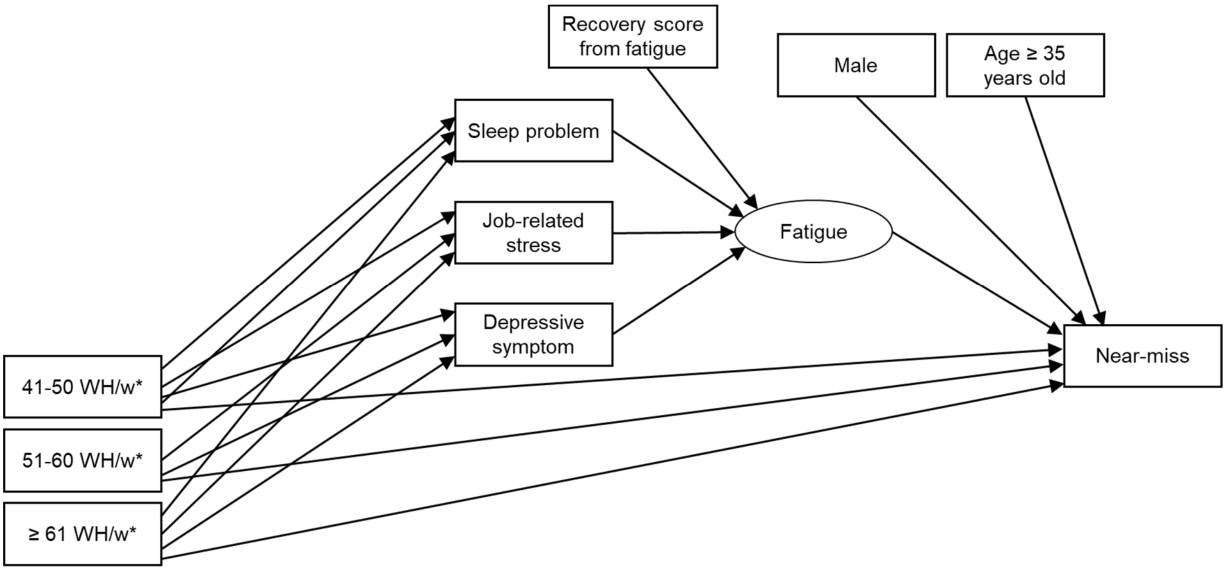

b: Model 3

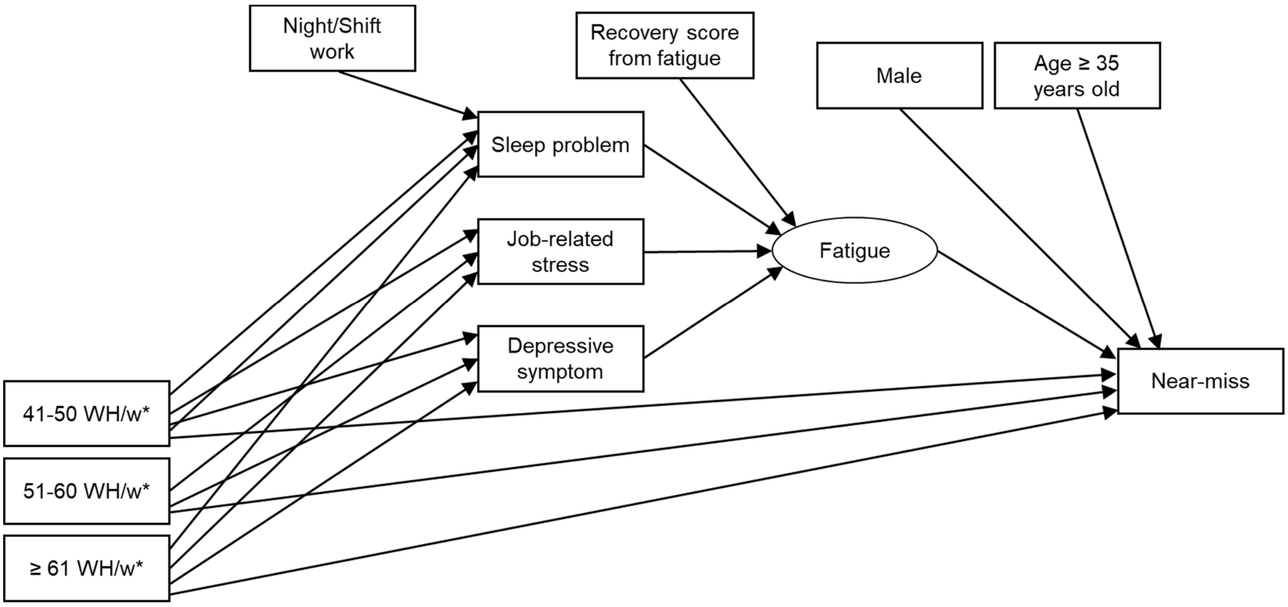

Figure S1: Structural equation model for relationship between near-misses and working hours in sensitivity analysis. a: Model 2 includes the recovery score from fatigue in the fatigue model. b: Model 3 includes the recovery score from fatigue in the fatigue model and night shift work in the sleep problem model. WH, working hours; w, week. * The reference category is 35–40 WH/w. “41–50 WH/w,” “51–60 WH/w,” “≥ 61 WH/w,” “Male,” “Age ≥ 35 years old,” and “Night/Shift work” are dummy variables, coded 1 if applicable and 0 otherwise; the circle represents a latent variable.
