# Supplementary material for: A Generalized Structural Equation Model Approach to Long Working Hours and Near-Misses among Healthcare Professionals in Japan
Source: Int J Environ Res Public Health. 2021 Jul 4;18(13):7154. doi: 10.3390/ijerph18137154 (PMC8296918; doi:10.3390/ijerph18137154)
Supplement: Supplementary file 1 [file ijerph-18-07154-s001.zip › supp_table_p.pdf]

SUPPLEMENTARY MATERIALS

Table S1. Direct, indirect, and total effects of working hours on near-misses during the past 6 months among healthcare professionals for sensitivity analysis

	n/Number of Near-Misses	Direct Effect	Indirect Effect			Total Effect (Direct + Indirect)
		Working Hour → Near-Miss	Working Hour → Job-Related Stress → Fatigue → Near-Miss	Working Hour →Sleep Problem → Fatigue → Near-Miss	Working Hour →Depressive Symptom → Fatigue → Near-Miss	
		OR (95%CI)	OR (95%CI)	OR (95%CI)	OR (95%CI)	OR (95%CI)
Model 2 in Supplemental Figure 1						
Working hours/week						
35-40 h/w	507/231	1.00 (reference)	1.00 (reference)	1.00 (reference)	1.00 (reference)	1.00 (reference)
41-50 h/w	665/338	1.13 (0.87, 1.44)	1.14 (1.08, 1.23)	1.02 (0.98, 1.05)	1.00 (0.99, 1.01)	1.30 (1.01, 1.70)
51-60 h/w	170/92	1.12 (0.79, 1.66)	1.28 (1.16, 1.45)	1.05 (1.00, 1.12)	1.01 (0.98, 1.03)	1.52 (1.05, 2.25)
≥61 h/w	148/80	1.09 (0.73, 1.59)	1.36 (1.21, 1.56)	1.08 (1.02, 1.16)	1.01 (0.96, 1.08)	1.61 (1.06, 2.39)
Model 3 in Supplemental Figure 1						
Working hours/week						
35-40 h/w	507/231	1.00 (reference)	1.00 (reference)	1.00 (reference)	1.00 (reference)	1.00 (reference)
41-50 h/w	665/338	1.13 (0.87, 1.44)	1.14 (1.08, 1.23)	1.01 (0.98, 1.05)	1.00 (0.99, 1.01)	1.30 (1.01, 1.69)
51-60 h/w	170/92	1.12 (0.78, 1.66)	1.28 (1.16, 1.46)	1.04 (0.99, 1.11)	1.01 (0.98, 1.03)	1.50 (1.04, 2.22)
≥61 h/w	148/80	1.09 (0.73, 1.59)	1.36 (1.21, 1.56)	1.07 (1.01, 1.14)	1.01 (0.96, 1.08)	1.59 (1.05, 2.36)

OR, odds ratio; 95% CI, 95% confidence interval; h/w, hours per week

Table S2. Direct, indirect, and total effects of working hours on near-misses during the past 6 months in healthcare professionals for model including relationship among the mediated variables.

	n / Number of Near-Misses	Direct Effect	Indirect Effect					Total Effect (Direct + Indirect)
		Working Hour → Near Miss	Working Hour → Job-Related Stress → Fatigue → Near Miss	Working Hour →Sleep Problem → Fatigue → Near Miss	Working Hour →Depressive Symptom → Fatigue → Near Miss	Working Hour → Job-Related Stress →Sleep Problem → Fatigue → Near Miss	Working Hour → Job-Related Stress →Depressive Symptom → Fatigue → Near Miss	
		OR (95%CI)	OR (95%CI)	OR (95%CI)	OR (95%CI)	OR (95%CI)	OR (95%CI)	OR (95%CI)
Working hours/week								
35-40 h/w	507 / 231	1.00 (reference)	1.00 (reference)	1.00 (reference)	1.00 (reference)	1.00 (reference)	1.00 (reference)	1.00 (reference)
41-50 h/w	665 / 338	1.10 (0.85, 1.42)	1.12 (1.07, 1.21)	0.99 (0.96, 1.01)	1.00 (0.98, 1.02)	1.02 (1.01, 1.04)	1.00 (0.98, 1.02)	1.23 (0.97, 1.64)
51-60 h/w	170 / 92	1.11 (0.78, 1.63)	1.25 (1.14, 1.41)	1.00 (0.95, 1.04)	1.00 (0.98, 1.02)	1.04 (1.02, 1.08)	1.00 (0.96, 1.03)	1.38 (1.01, 2.12)
≥ 61 h/w	148 / 80	1.03 (0.69, 1.53)	1.31 (1.18, 1.53)	1.01 (0.96, 1.06)	1.00 (0.98, 1.02)	1.05 (1.02, 1.10)	1.00 (0.95, 1.04)	1.37 (0.96, 2.21)

OR, odds ratio; 95% CI, 95% confidence interval; h/w, hours per week
